# Reassessment of MxiH subunit orientation and fold within native *Shigella* T3SS needles using surface labelling and solid-state NMR

**DOI:** 10.1016/j.jsb.2015.10.005

**Published:** 2015-12

**Authors:** Joeri Verasdonck, Da-Kang Shen, Alexander Treadgold, Christopher Arthur, Anja Böckmann, Beat H. Meier, Ariel J. Blocker

**Affiliations:** a Physical Chemistry, ETH Zurich, 8093 Zurich, Switzerland; b School of Cellular & Molecular Medicine, University of Bristol, BS8 1TD Bristol, United Kingdom; c School of Chemistry, University of Bristol, BS8 1TS Bristol, United Kingdom; d Institut de Biologie et Chimie des Protéines, Bases Moléculaires et Structurales des Systèmes Infectieux, Labex Ecofect, UMR 5086 CNRS, Université de Lyon, Lyon, France; e Schools of Cellular & Molecular Medicine and Biochemistry, University of Bristol, BS8 1TD Bristol, United Kingdom

**Keywords:** CR, Congo red, *Shigella*, Type III secretion, Needle, Surface labelling, Solid-state NMR

## Abstract

T3SSs are essential virulence determinants of many Gram-negative bacteria, used to inject bacterial effectors of virulence into eukaryotic host cells. Their major extracellular portion, a ∼50 nm hollow, needle-like structure, is essential to host cell sensing and the conduit for effector secretion. It is formed of a small, conserved subunit arranged as a helical polymer. The structure of the subunit has been studied by electron cryomicroscopy within native polymers and by solid-state NMR in recombinant polymers, yielding two incompatible atomic models. To resolve this controversy, we re-examined the native polymer used for electron cryomicroscopy via surface labelling and solid-state NMR. Our data show the orientation and overall fold of the subunit within this polymer is as established by solid-state NMR for recombinant polymers.

## 1 Introduction

Type III secretion systems (T3SSs) are found in many Gram-negative bacteria, forming injection devices to deliver bacterial proteins into eukaryotic host cells during infection. Hence, understanding their three-dimensional structure is important for design of new broad-spectrum strategies to combat bacterial pathogens (Kosarewicz et al., 2012). These macromolecular assemblies are composed of ∼25 proteins and span the bacterial cytoplasm, membranes and the extracellular space to connect with the host cell plasma membrane. T3SSs are made up of three parts (Blocker et al., 1999): a cytoplasmic portion, a transmembrane region, and an extracellular ‘needle’. The inner membrane region houses the export apparatus, which connects to the periplasmic end of the needle, itself held within the central cavity of an outer membrane secretin (Hodgkinson et al., 2009). The secreted proteins pass into the host cell via a translocation pore assembled from the tip of the needle into the host membrane (Blocker et al., 1999). The needle-like structure is ∼50 nm long and 7 nm wide. It is hollowed by a central channel 15 Å in diameter (Fujii et al., 2012), which serves as a secretion conduit for effectors and is made by the helical assembly of a single, small, conserved protein (Blocker et al., 2001, Cordes et al., 2003).

*Shigella flexneri*, the agent of human bacillary dysentery, uses a T3SS for invasion of, and dissemination within, the gut epithelial lining (Schroeder and Hilbi, 2008). Within the *Shigella* needle, the MxiH subunit is arranged into a helical polymer that shares packing parameters with the extracellular portions of the bacterial flagellar filament (Cordes et al., 2003), to which T3SSs are evolutionarily related. The needle is topped by a “tip complex”, which is the host-cell sensor and eventually forms the translocation pore (Cheung et al., 2015, Mueller et al., 2005, Veenendaal et al., 2007). Point mutations in the needle protein lead to deregulated secretion as well as functionally altered tips (Kenjale et al., 2005, Torruellas et al., 2005, Veenendaal et al., 2007), indicating it is directly involved in transducing the signal of host cell contact to the base of the apparatus (Kenjale et al., 2005, Torruellas et al., 2005). This and the fact that it is a major extracellular component of T3SSs justify the interest in understanding its structure.

Following discovery that short C-terminal deletions prevent MxiH polymerization, partial crystal and NMR structures of monomers from several species revealed a helix-turn-helix hairpin fold (Blocker et al., 2008, Deane et al., 2006, Poyraz et al., 2010). In all these, the terminal half of the N-terminal α-helix is unstructured and invisible to various degrees. The *S. flexneri* pseudoatomic needle architecture was initially modelled by docking MxiH_CΔ5_ crystal monomers into a 16 Å-resolution electron density map of the natively polymerized needle obtained using negative stain electron microscopy (Cordes et al., 2003). Only one of the two crystallographic forms fitted into that map and it did so in only one orientation (Deane et al., 2006), leading to the N-terminus of MxiH facing the inner side of the channel in this initial model (Deane et al., 2006). This subunit orientation was also used in several subsequent models built using solution-state NMR structures (Chatterjee et al., 2011, Rathinavelan et al., 2010, Wang et al., 2010, Wang et al., 2007, Zhang et al., 2006, Zhang et al., 2007) and in a refined model based on a 7.7-Å electron density map obtained using electron cryomicroscopy (cryoEM), where subunit orientation was also assessed using 1–3 amino acid N and C-terminal deletions (Fujii et al., 2012).

However, using solid-state NMR, an atomic-resolution model of the recombinantly generated *Salmonella enterica* serovar Typhimurium needle, composed of PrgI, which is a homologue of MxiH, was determined and the N-terminus was found on the outside face of the needle (Demers et al., 2014, Demers et al., 2013, Loquet et al., 2013a, Loquet et al., 2012a). The outside orientation of the N-terminus was confirmed by immunogold labelling of both *Salmonella typhimurium* and *S. flexneri* needles generated by overexpression of their component subunits with 10–15 amino acid N-terminal tags. Subsequently, solid-state NMR measurements of wild-type *S. flexneri* serotype 6 needles, also polymerized *in vitro*, showed its MxiH subunit shared very similar secondary structure elements to those defined for PrgI using the same method (Demers et al., 2013). The N-terminus of the subunit in this polymer was also confirmed to lie on the outside using identical immunolabeling methods. Therefore, an atomic model of *S. flexneri* needles was built by homology to that calculated for *Salmonella* needles. However, the cryoEM and solid-state NMR models differ not only in subunit orientation but also in polypeptide chain location at their surface. Indeed a short “protrusion” is seen within the cryoEM map that is absent or different in each available solid-state NMR model (Demers et al., 2014, Demers et al., 2013, Loquet et al., 2012a). This is why the *Shigella* solid-state NMR model was then recalculated whilst simultaneously imposing constraints derived from the *Shigella* cryoEM map (Demers et al., 2014, Loquet et al., 2012b). However, even in this new model, the N-terminus of the subunit does not fit fully into the protrusion density. This led us to wonder if the native and recombinant polymers might not differ, even though they share similar numbers of subunits per helical turn (Loquet et al., 2013b). We here investigate *in vivo* assembled MxiH needles from *S. flexneri* serotype 5 shaved from the bacterial surface, as used in cryoEM (Fujii et al., 2012), using surface labelling and solid-state NMR and find that they form virtually identical structures to those established by solid-state NMR on recombinant *Shigella* needles.

## 2 Materials and methods

### 2.1 Bacterial strains and cell culture

All bacterial strains used in this study are listed in Supplementary Table S1. *S. flexneri* strains were maintained and selected on CR agar plates (Meitert et al., 1991), and grown at 37 °C in trypticase soy broth (Becton Dickinson) supplemented with antibiotics when necessary (100 μg of ampicillin ml^−1^, 50 μg of kanamycin ml^−1^, 20 μg of chloramphenicol ml^−1^; Sigma).

### 2.2 Cloning and mutagenesis of *mxiH*

The DNA sequence of *mxiH*_L32C_ was amplified by two-step PCR using the primers listed in Supplementary Table S2, with modification of T95G and G96T, whilst *mxiH*_V68C_ was synthesized by Eurofins, with modification of G202T, T203G and G204T. Both constructs were cloned into vector pACT3 via NdeI and HindIII (Shen et al., 2010) and verified by commercial sequencing (Eurofins).

### 2.3 Analysis of protein secretion

Leakage of the Ipa proteins and CR-induced protein secretion were examined as previously described (Martinez-Argudo and Blocker, 2010).

### 2.4 Needle preparations for surface labelling

Needles were purified as described previously (Cordes et al., 2005), but using 200 μM IPTG (isopropyl-β-d-thiogalactopyranoside, Sigma) to induce *mxiH* expression from pACT3*mxiH* (Shen et al., 2010). For wild-type and MxiH_L32C_, 500 ml of trypticase soy broth culture were grown for needle preparation. However, due to the much lower yield of MxiH_V68C_, 2–3 L of this strain were grown to obtain about 250 μg of needles.

### 2.5 Needle preparations for solid-state NMR

For this purpose, needles were obtained in batches from about 20 L total of bacteria growing in M9-type minimal media (Studier, 2005) supplemented with 2 g ml^−1^ ^15^N labelled ammonium chloride and U–^13^C_6_ labelled d-glucose (Cambridge Isotope Laboratories/CK Gas). Overnight cultures of 5 ml M9 minimal Media with labelled glucose and NH_4_Cl, were inoculated with Δ*mxiH* (pACT3*mxiH*; (Shen et al., 2010)), with kanamycin and chloramphenicol. The cultures were incubated at 37 °C with shaking at 180 rpm. After 16 hrs, the optical density at 600 nm (OD_600_) was measured. If the OD_600_ was >1.00, all of the 5 ml culture was used to inoculate 1 L of M9 minimal media, labelled as above, in a 5 L sterile conical flask, with 200 μM IPTG and incubated at 37 °C with shaking at 180 rpm. After 16 hrs, the OD_600_ was measured again, if OD_600_ was >1.10, growth was halted and needles purified as previously described (Cordes et al., 2005, Fujii et al., 2012). The final pellet was resuspended in 0.01% of the initial culture volume in sterile 20 mM Tris–HCl pH 7.4, 100 mM NaCl, 10% w/v d-(+)-Trehalose (Sigma) and 0.02% w/v sodium azide, generally attaining 3–7 mg ml^−1^ in protein concentration, and then flash frozen in liquid nitrogen and stored at −80 °C until use. From 20 L of bacterial culture about 7 mg of labelled needles were obtained.

### 2.6 Electron microscopy

Appropriately diluted needles (about 0.1 μg ml^−1^) were deposited onto 300-mesh, freshly glow-discharged, Formvar and carbon-coated copper grids, and subsequently stained for 1 min with 1% (w/v) phosphotungstic acid at pH 7. Needles were visualized in a Tecnai 12 transmission electron microscope (FEI) fitted with an FEI Eagle 4 k × 4 k CCD camera at ×20,000 magnification using FEI Tecnai Imaging Analysis software.

### 2.7 PEG maleimide-labelling and analysis

25 μl of needles of 1 mg ml^−1^ in PBS pH 6.6 were used for the cysteine modification by the thiol-specific reagent methoxypolyethylene glycol 5000 maleimide (PEG maleimide; Fluka) as previously described (Hara et al., 2012). Briefly, 2.5 μl of PEG maleimide at 100 mg ml^−1^ was added into needles solution, mixed and incubated at 37 °C for 30 min with shaking. 2.5 μl of β-mercaptoethanol was then added to quench the reaction. Samples were centrifuged at 100,000*g* for 30 min at 4 °C and about 30 μl supernatant were collected and mixed with 10 μl of 4 × SDS loading buffer. The pellets were re-suspended into 40 μl of 1× SDS loading buffer. All samples were boiled at 100 °C for 10 min and an aliquot of 10 μl was used for Western blot analysis. Proteins separated by SDS–PAGE were transferred onto PVDF membrane (Immobilon FL, Millipore), hybridized with anti-MxiH serum (Magdalena et al., 2002) and then a goat anti-rabbit IgG Alexa 680 (Invitrogen) secondary antibody conjugate. The membranes were visualized using an Odyssey infrared imaging system (LICOR Biosciences).

### 2.8 Cy3 maleimide-labelling and mass spectrometry analysis

To minimize oxidation of thiols, 150–250 μl of needles of 1 mg ml^−1^ in PBS pH 6.6 were treated with 3 mg ml^−1^ (final concentration) Tris (carboxyethyl) phosphine (TCEP, Sigma) at room temperature for 10 min within an anaerobic hood. Needles were collected by ultracentrifugation (15 min, 385,840*g*, 4 °C; TLA100; Beckman-Coulter), resuspend in 100 μl of degassed PBS pH 6.6 containing one pack of Cy3 maleimide (GE Healthcare, PA23031) and incubated for 1 hr within the anaerobic hood at room temperature. After removal of the supernatants by ultracentrifugation, needles were gently washed twice further via repeated centrifugation, and finally resuspended in 100 μl of ammonium acetate (100 mM, pH 6.6) and then ultracentrifuged. The pellets were resuspended in 6–10 μl of ammonium acetate for mass spectrometry analysis. Samples were analysed by electrospray mass spectrometry on an Orbitrap Elite Mass Spectrometer. Samples were directly infused by syringe pump and analysed in positive ion mode over a mass to charge range of 600–2000 Da, with a resolution of 60,000. Raw electrospray charge envelopes were deconvoluted using MagTran (Zhang and Marshall, 1998).

### 2.9 Solid-state NMR

Five to seven mg of uniformly ^13^C,^15^N labelled natively grown needles were filled into a ZrO_2_ 1.9 mm rotor (Bruker) using a homemade filling device (Böckmann et al., 2009) spun at 35,000 rpm for 3 h at 5 °C in an SW40 TI rotor and an optima L90-K ultracentrifuge (Beckman). A 20 ms DARR of the natively grown needles was recorded and assigned using the assignment of the *in vitro* polymerized needles by Demers et al. as a template (Demers et al., 2013). The residues conserved between the two serotypes could be identified and assigned in this way for the 20 ms DARR and an NCA spectrum. 3D NCACB and CCC, as well as 200 ms DARR spectra were recorded to assign the non-conserved residues. All spectra were recorded at a 20.0 T static field at 270 K at 10 °C and an 18 kHz MAS frequency. Spectra were processed using Topspin 3.2 (Bruker BioSpin) and a shifted (2–3) cosine squared apodization function was used as a window function for all spectra. An overview of the experimental details can be found in Supplementary Table S3. Assignments (Supplementary Table S4) were preformed using CcpNmr Analysis (Stevens et al., 2011) and deposited in BMRB under accession number 26614.

## 3 Results and discussion

### 3.1 MxiH cysteine point mutants behave functionally like wild-type

**Fig. 1:**
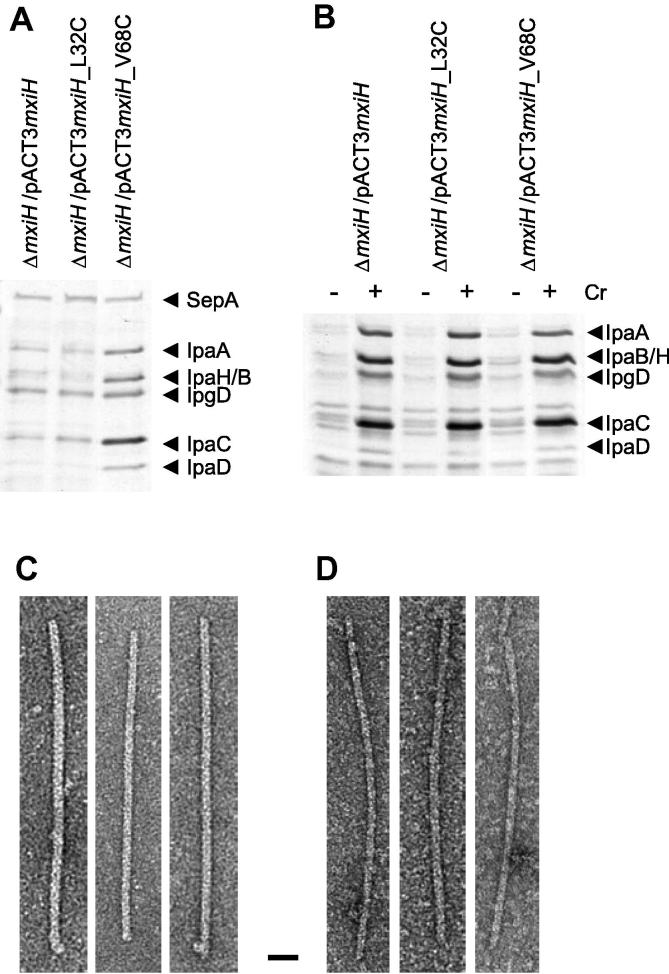
*mxiH* cysteine point mutants have normal secretion characteristics and form morphologically normal needles. (A) Exponential leakage of the Ipa proteins and (B) induced secretion of Ipa proteins after the addition (+) or not (−) of Congo red (Cr) were analysed by silver staining after SDS–PAGE. The positions of the major Ipa proteins detected by silver staining are indicated on the left side. (C and D) Electron micrographs of purified superlong MxiH_L32C_ and MxiH_V68C_ needles, negatively stained. Bar equals 20 nm. The data shown here are representative of two independent experiments giving similar results.

To determine the orientation of termini of the MxiH subunit, we constructed two mutants with single cysteine (C) substitutions at N-terminal leucine 32 (L32C) and C-terminal valine 68 (V68C). The differing locations of these amino acids in the models of Fujii et al. (2012) and Demers et al. (2014) is shown in Supplementary Fig. S1. We first verified the functionality of these strains by checking their ability to perform T3SS-mediated Ipa protein “leakage” and “Congo red (CR) induction” in comparison with the wild-type strain (WT). “Leakage” is a slow, low-level Ipa protein secretion whereby around 5% of Ipa proteins are secreted (Magdalena et al., 2002), indicating a functional type III secretion apparatus; whilst “CR Induction” describes the burst of Ipa protein secretion upon the addition of CR (Bahrani et al., 1997), which is an artificial inducer of the *Shigella* T3SS. With respect to “leakage” (Fig. 1A) and “CR induction”, both strains behaved like the WT (Fig. 1B). This indicates that both mutants are able to form functional needles and tip complexes, which are required for leakage and CR induction (Kenjale et al., 2005, Veenendaal et al., 2007), respectively.

We next checked for superlong needle formation via overexpression of MxiH_L32C_ and MxiH_V68C_, a property of wild-type needles (Cordes et al., 2003, Tamano et al., 2000), which was vital to successful surface labelling. Examination of purified needle preparation by negative-electron microscopy showed that both constructs produce longer needles (Fig. 1C and D), though the yield of MxiH_V68C_ is about 4 times lower than that of MxiH_L32C_, which is itself about 2 times lower than that of WT MxiH. Together, these data indicate that, both MxiH_L32C_ and MxiH_V68C_ have WT-like secretion profiles and can form morphologically normal needles.

### 3.2 The N-terminus of native MxiH is located on the outside of the needle

To determine the orientation of termini of the MxiH subunit, purified needles were labelled with both Cy3-maleimide and PEG-maleimide. The assumption was that the >5 kDa PEG-maleimide would not be able to penetrate the inside the needle channel, whilst the ∼700 Da Cy3-maleimide might.

**Fig. 2:**
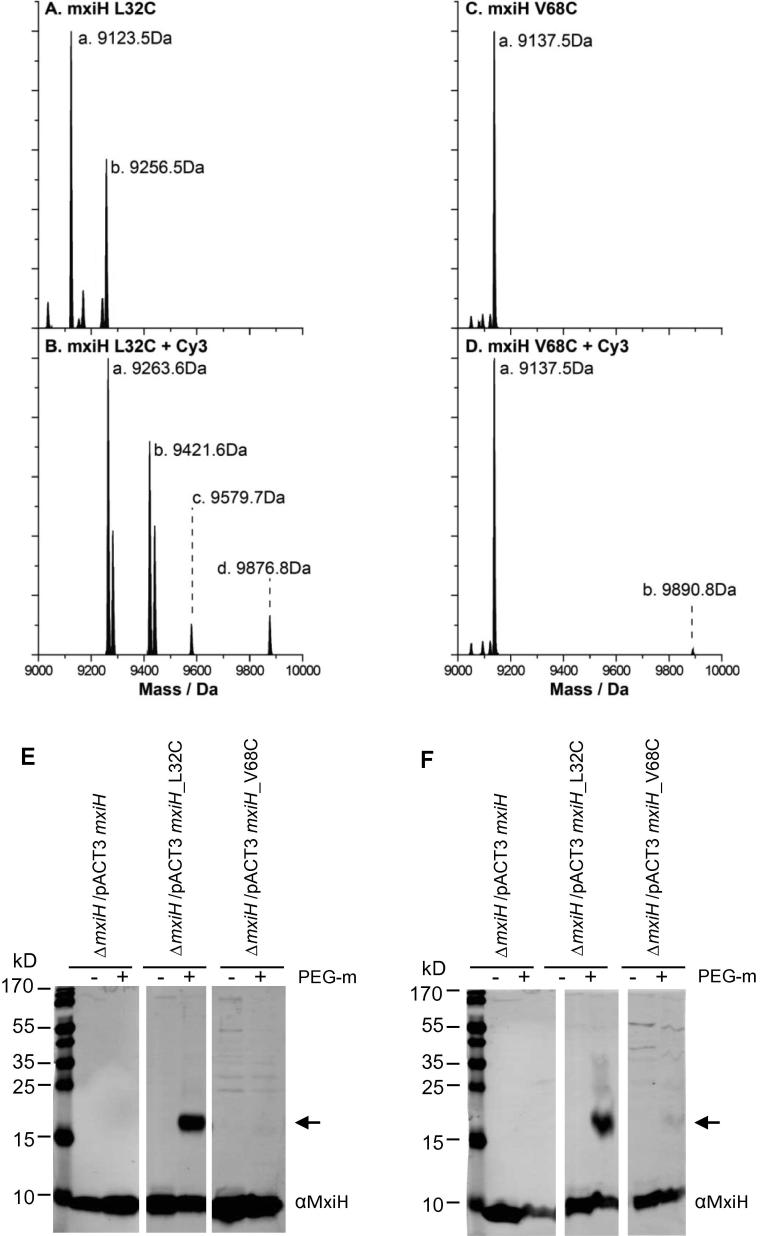
MxiH_L32C_ is located on the outside of the needle. Native mass spectrometry analysis of purified needles (A and C) and purified needles labelled with Cy3 maleimide (B and D). These spectra were deconvoluted from those shown in Supplementary Fig. S2 to calculate the mass of the constituents in the mass window of 9000–10,000 daltons (Da), which covers the masses of the expected products. Masses are indicated in Da. Intensity is indicated in arbitrary units. (E–F) Immunoblotting of purified needles exposed (+) or not (−) to PEG5000 maleimide (PEG-m); (E) pellets; (F) supernatants. The antibody used for the Western blots is indicated on the right. The arrow shows MxiH covalently coupled to PEG5000 maleimide. Data shown are representative of 2 independent experiments giving similar results.

For the Cy3 maleimide experiments, electrospray mass spectra of both untreated and labelled needles were recorded over mass-to-charge ratio (m/z) window of 600–2000 (Supplementary Fig. S2). These spectra were deconvoluted to calculate the mass of the constituents in the mass window of 9000–10,000 Da, which covers the masses of the expected products. The peak at 9123.5 Da corresponds to native MxiH_L32C_ (Fig. 2A) and this peak was totally absent from same needles labelled with Cy3 maleimide, but new peaks appeared 9263.6 Da, the calculated mass of MxiH_L32C_ + maleimide, and 9876.8 Da, the mass of MxiH_L32C_ + Cy3 maleimide (Fig. 2B), indicating all available MxiH_L32C_ had been labelled by Cy3-maleimide but that, for unknown reasons, the majority of the Cy3 had been hydrolysed away from the coupled maleimide during the experimental procedure.

In contrast, the peak at 9137.5 Da corresponds to native MxiH_V68C_ (Fig. 2C) and it is always there, even when needles have been exposed to Cy3-maleimide. The peak at 9890.8 Da corresponds to the mass of MxiH_V68C_ + Cy3, however, its intensity is only about 2% of that of unlabelled MxiH_V68C_ (Fig. 2D), whereas 100% of MxiH_L32C_ had been labelled since the unlabelled peak is completely lost (Fig. 2B). This supports the notion that amino acid 68 of MxiH is normally hardly accessible to reagents added to the solution bathing needles, whereas amino acid 32 is highly accessible to such reagents. This suggests that amino 32 lies on the needle surface and Cy3-maleimide was unable to diffuse efficiently into the channel that exists in the needle polymer.

For the PEG-maleimide experiments, both native and labelled needles, including intact ones, pelleted via high-speed centrifugation, and potentially disassembled subunits or very short fragments, remaining in the supernatants, were analysed by Western blotting using anti-MxiH antibodies. For intact needles, an ∼5-kDa shift, corresponding to the size of PEG-maleimide, was observed for MxiH_L32C_, but not from either MxiH or MxiH_V68C_. For disassembled needles, some trace of a 5-kDa shift was also observed for MxiH_V68C_ but not for MxiH. Taken together, these data on the accessibility of L32C but not V68C, to both Cy3-maleimide and PEG-maleimide, indicate that the N-terminus of the MxiH is exposed on the outside of native needles, thereby invalidating the model of Fujii et al. (2012).

### 3.3 Native and recombinant needles share virtually the same structure

**Fig. 3:**
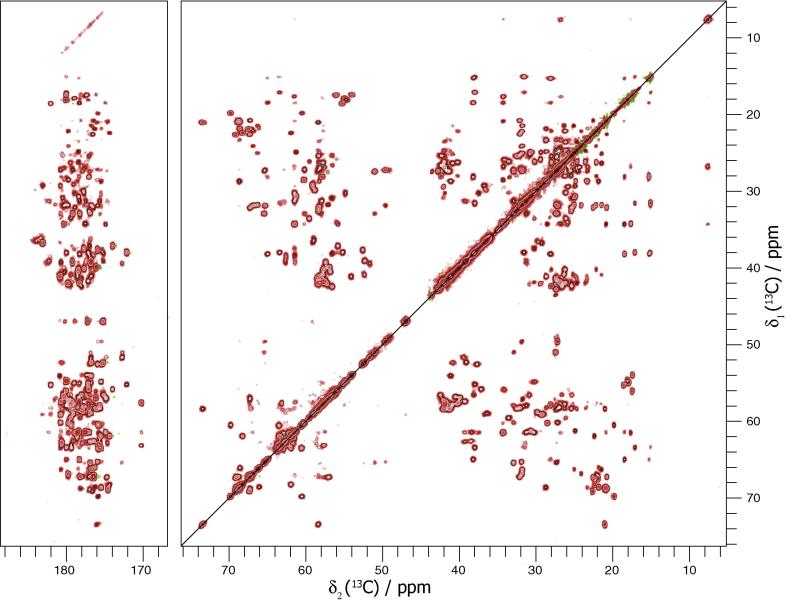
20-ms DARR spectrum of the natively grown uniformly-labelled MxiH needles. The data has been zero-filled and apodized with a shifted squared sine-bell function. The lowest contour level is plotted at 4.4 times the noise RMSD. A version of this figure where assigned peaks have been labelled on one side of the diagonal is available in Supplemental Fig. S4.

**Fig. 4:**
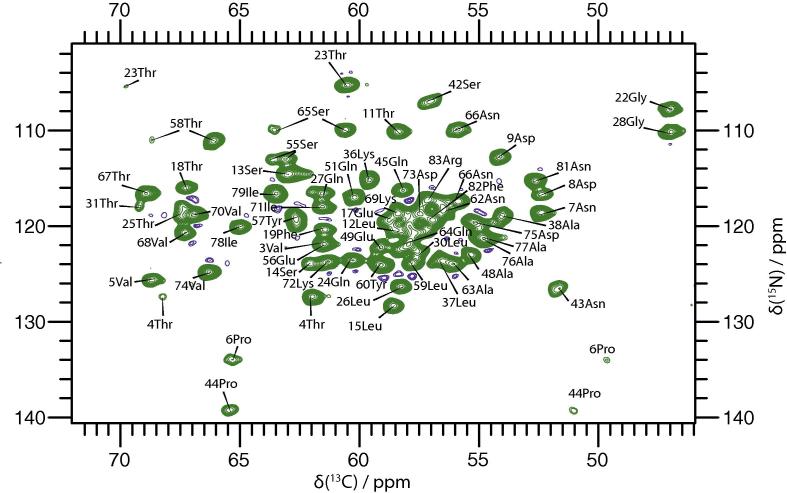
NCA spectrum of uniformly ^13^C,^15^N-labelled natively grown MxiH needles. The data was recorded at a 20.0 T static magnetic field and the data was zero-filled and apodized in both dimensions with a shifted squared sine-bell function. Assigned peaks are labelled. The lowest contour level is plotted at 4.7 times the noise RMSD.

**Fig. 5:**
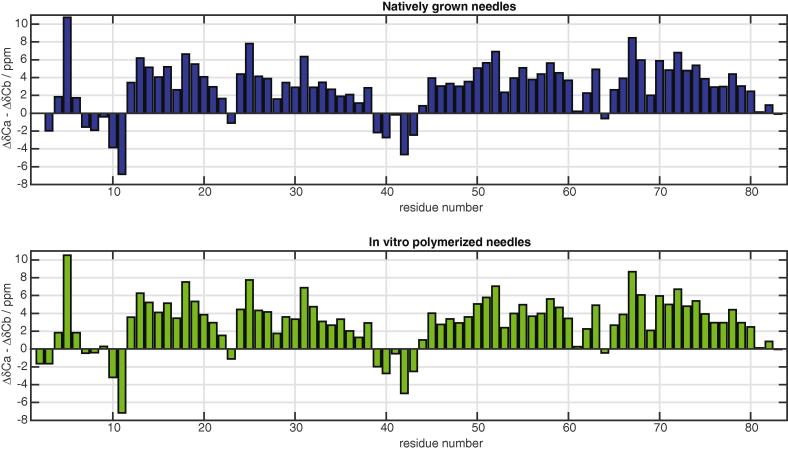
Secondary chemical shifts of the natively grown MxiH needles and *in vitro* polymerized MxiH needles. Former are shown at the *top*, whilst latter, i.e. that of Demers et al. (2013), are reproduced at the *bottom*.

The 20 ms DARR (Fig. 3) solid-state NMR spectrum of the natively grown needles strongly resembles the 50 ms PDSD spectrum of the *in vitro* polymerized needles by Demers et al. (2013), with some pronounced differences observed as a result of sequence variations between the two different serotypes used (Supplemental Fig. S3). We observe line widths in the order of 90 Hz, which corresponds to 0.42 ppm on an 850 MHz spectrometer. Demers et al. observe line widths between 0.09 and 0.25 ppm for [1-^13^C]glucose and [2-^13^C]glucose labelled samples. The difference in line widths can be explained by the different ^13^C labelling of the samples with the sparse labelling avoiding most ^13^C–^13^C one-bond J couplings, which are an important broadening mechanism. It is therefore reasonable to assume that the local order of the sample is similar for both preparation methods. For the assignment of native needles the assignment by Demers et al. used as a template for the 20 ms DARR and NCA (Fig. 4 and Supplemental Fig. S4) spectra could largely be taken over. Spin systems corresponding to the seven mutation sites between the two serotypes were all identified in a NCACB spectrum (Supplementary Fig. S5). Some of these spin systems could by unambiguously typed and assigned to a specific residue. For the remainder, the Cα and Cβ resonances of the spin systems was correlated with the sequential neighbours using a 2D DARR (200 ms, Supplemental Fig. S6) and CCC (with an 80 ms DARR mixing element, Supplemental Fig. S7) to obtain the sequence-specific assignment, including the ^15^N frequency. To assign the remaining spin systems to their respective residues, a 200 ms DARR (Supplemental Fig. S6) and a CCC spectrum (Supplemental Fig. S7) were recorded. Sequential contacts can be observed in these spectra, thereby allowing the proper placement of the spin systems in the sequence. In total, 97% of backbone ^13^C and 93% of backbone ^15^N was assigned. A direct comparison of the chemical shifts for N, Cα, Cβ and C’ can be found in the Supplemental Information (Supplemental Fig. S8). The secondary chemical shifts of this assignment and those of Demers et al. are nearly identical throughout the entire sequence, even where the serotype mutations occur (Fig. 5). This is a strong indication that, natively grown and *in vitro* polymerized needles have the same monomer structure and, very likely, the same supramolecular packing.

## 4 Conclusion

Using native needles, as in the cryoEM experiments (Fujii et al., 2012), we have reassessed, using different methods, the subunit orientation with native MxiH needles and unambiguously find the N-terminus to be facing the outside of the polymer. We have been able to produce labelled needles natively from *Shigella* in sufficient amounts for solid-state NMR and found that these are highly ordered and hence generate well-resolved spectra. Assignment of the secondary chemical shifts within native MxiH needles show that they are highly similar to those of Demers et al. (2013) obtained from recombinant material, throughout the entire sequence and even where serotype-specific sequence variation occurs. Therefore, the secondary structure of both proteins within the respective native and recombinant polymers is nearly identical, as are mass/length and handedness of both polymers (Deane et al., 2006, Hodgkinson et al., 2009, Loquet et al., 2013b). This suggests the tertiary and quaternary packing of both subunits is also very similar, justifying the combination of the extensive NMR data recorded on recombinant needles with cryoEM data from native needles. The remaining, possibly functionally relevant, differences between the cryoEM map and the NMR structures, e.g. at the protrusion site, are therefore probably due to experimental imprecisions.

## References

[b0005] Bahrani F.K., Sansonetti P.J., Parsot C. (1997). Secretion of Ipa proteins by *Shigella flexneri*: inducer molecules and kinetics of activation. Infect. Immun..

[b0010] Blocker A., Gounon P., Larquet E., Niebuhr K., Cabiaux V., Parsot C., Sansonetti P. (1999). The tripartite type III secretion of *Shigella flexneri* inserts IpaB and IpaC into host membranes. J. Cell Biol..

[b0015] Blocker A., Jouihri N., Larquet E., Gounon P., Ebel F., Parsot C., Sansonetti P., Allaoui A. (2001). Structure and composition of the *Shigella flexneri* “needle complex”, a part of its type III secretion. Mol. Microbiol..

[b0020] Blocker A.J., Deane J.E., Veenendaal A.K., Roversi P., Hodgkinson J.L., Johnson S., Lea S.M. (2008). What’s the point of the type III secretion system needle?. Proc. Natl. Acad. Sci. U.S.A..

[b0025] Böckmann A., Gardiennet C., Verel R., Hunkeler A., Loquet A., Pintacuda G., Emsley L., Meier B.H., Lesage A. (2009). Characterization of different water pools in solid-state NMR protein samples. J. Biomol. NMR.

[b0030] Chatterjee S., Zhong D., Nordhues B.A., Battaile K.P., Lovell S., De Guzman R.N. (2011). The crystal structures of the Salmonella type III secretion system tip protein SipD in complex with deoxycholate and chenodeoxycholate. Protein Sci..

[b0035] Cheung M., Shen D.K., Makino F., Kato T., Roehrich A.D., Martinez-Argudo I., Walker M.L., Murillo I., Liu X., Pain M., Brown J., Frazer G., Mantell J., Mina P., Todd T., Sessions R.B., Namba K., Blocker A.J. (2015). Three-dimensional electron microscopy reconstruction and cysteine-mediated crosslinking provide a model of the type III secretion system needle tip complex. Mol. Microbiol..

[b0040] Cordes F.S., Komoriya K., Larquet E., Yang S., Egelman E.H., Blocker A., Lea S.M. (2003). Helical structure of the needle of the type III secretion system of *Shigella flexneri*. J. Biol. Chem..

[b0045] Cordes F.S., Daniell S., Kenjale R., Saurya S., Picking W.L., Picking W.D., Booy F., Lea S.M., Blocker A. (2005). Helical packing of needles from functionally altered Shigella type III secretion systems. J. Mol. Biol..

[b0050] Deane J.E., Roversi P., Cordes F.S., Johnson S., Kenjale R., Daniell S., Booy F., Picking W.D., Picking W.L., Blocker A.J., Lea S.M. (2006). Molecular model of a type III secretion system needle: implications for host–cell sensing. Proc. Natl. Acad. Sci. U.S.A..

[b0055] Demers J.P., Sgourakis N.G., Gupta R., Loquet A., Giller K., Riedel D., Laube B., Kolbe M., Baker D., Becker S., Lange A. (2013). The common structural architecture of *Shigella flexneri* and *Salmonella typhimurium* type three secretion needles. PLoS Pathog..

[b0060] Demers J.P., Habenstein B., Loquet A., Kumar Vasa S., Giller K., Becker S., Baker D., Lange A., Sgourakis N.G. (2014). High-resolution structure of the Shigella type-III secretion needle by solid-state NMR and cryo-electron microscopy. Nat. Commun..

[b0065] Fujii T., Cheung M., Blanco A., Kato T., Blocker A.J., Namba K. (2012). Structure of a type III secretion needle at 7-A resolution provides insights into its assembly and signaling mechanisms. Proc. Natl. Acad. Sci. U.S.A..

[b0070] Hara N., Morimoto Y.V., Kawamoto A., Namba K., Minamino T. (2012). Interaction of the extreme N-terminal region of FliH with FlhA is required for efficient bacterial flagellar protein export. J. Bacteriol..

[b0075] Hodgkinson J.L., Horsley A., Stabat D., Simon M., Johnson S., da Fonseca P.C., Morris E.P., Wall J.S., Lea S.M., Blocker A.J. (2009). Three-dimensional reconstruction of the *Shigella* T3SS transmembrane regions reveals 12-fold symmetry and novel features throughout. Nat. Struct. Mol. Biol..

[b0080] Kenjale R., Wilson J., Zenk S.F., Saurya S., Picking W.L., Picking W.D., Blocker A. (2005). The needle component of the type III secretion of Shigella regulates the activity of the secretion apparatus. J. Biol. Chem..

[b0085] Kosarewicz A., Konigsmaier L., Marlovits T.C. (2012). The blueprint of the type-3 injectisome. Philos. Trans. R. Soc. Lond. B Biol. Sci..

[b0090] Loquet A., Sgourakis N.G., Gupta R., Giller K., Riedel D., Goosmann C., Griesinger C., Kolbe M., Baker D., Becker S., Lange A. (2012). Atomic model of the type III secretion system needle. Nature.

[b0095] Loquet A., Sgourakis N.G., Gupta R., Giller K., Riedel D., Goosmann C., Griesinger C., Kolbe M., Baker D., Becker S., Lange A. (2012). Corrigendum: atomic model of the type III secretion system needle. Nature.

[b0100] Loquet A., Habenstein B., Lange A. (2013). Structural investigations of molecular machines by solid-state NMR. Acc. Chem. Res..

[b0105] Loquet A., Habenstein B., Chevelkov V., Vasa S.K., Giller K., Becker S., Lange A. (2013). Atomic structure and handedness of the building block of a biological assembly. J. Am. Chem. Soc..

[b0110] Magdalena J., Hachani A., Chamekh M., Jouihri N., Gounon P., Blocker A., Allaoui A. (2002). Spa32 regulates a switch in substrate specificity of the type III secretion of *Shigella flexneri* from needle components to Ipa proteins. J. Bacteriol..

[b0115] Martinez-Argudo I., Blocker A.J. (2010). The Shigella T3SS needle transmits a signal for MxiC release, which controls secretion of effectors. Mol. Microbiol..

[b0120] Meitert T., Pencu E., Ciudin L., Tonciu M., Mihai I., Nicolescu S. (1991). Correlation between Congo red binding as virulence marker in Shigella species and Sereny test. Roum. Arch. Microbiol. Immunol..

[b0125] Mueller C.A., Broz P., Muller S.A., Ringler P., Erne-Brand F., Sorg I., Kuhn M., Engel A., Cornelis G.R. (2005). The V-antigen of Yersinia forms a distinct structure at the tip of injectisome needles. Science.

[b0130] Poyraz O., Schmidt H., Seidel K., Delissen F., Ader C., Tenenboim H., Goosmann C., Laube B., Thunemann A.F., Zychlinsky A., Baldus M., Lange A., Griesinger C., Kolbe M. (2010). Protein refolding is required for assembly of the type three secretion needle. Nat. Struct. Mol. Biol..

[b0135] Rathinavelan T., Zhang L., Picking W.L., Weis D.D., De Guzman R.N., Im W. (2010). A repulsive electrostatic mechanism for protein export through the type III secretion apparatus. Biophys. J..

[b0140] Schroeder G.N., Hilbi H. (2008). Molecular pathogenesis of *Shigella* spp.: controlling host cell signaling, invasion, and death by type III secretion. Clin. Microbiol. Rev..

[b0145] Shen D.K., Saurya S., Wagner C., Nishioka H., Blocker A.J. (2010). Domains of the *Shigella flexneri* type III secretion system IpaB protein involved in secretion regulation. Infect. Immun..

[b0150] Stevens T.J., Fogh R.H., Boucher W., Higman V.A., Eisenmenger F., Bardiaux B., van Rossum B.J., Oschkinat H., Laue E.D. (2011). A software framework for analysing solid-state MAS NMR data. J. Biomol. NMR.

[b0155] Studier F.W. (2005). Protein production by auto-induction in high density shaking cultures. Protein Expr. Purif..

[b0160] Tamano K., Aizawa S., Katayama E., Nonaka T., Imajoh-Ohmi S., Kuwae A., Nagai S., Sasakawa C. (2000). Supramolecular structure of the Shigella type III secretion machinery: the needle part is changeable in length and essential for delivery of effectors. EMBO J..

[b0165] Torruellas J., Jackson M.W., Pennock J.W., Plano G.V. (2005). The *Yersinia pestis* type III secretion needle plays a role in the regulation of Yop secretion. Mol. Microbiol..

[b0170] Veenendaal A.K., Hodgkinson J.L., Schwarzer L., Stabat D., Zenk S.F., Blocker A.J. (2007). The type III secretion system needle tip complex mediates host cell sensing and translocon insertion. Mol. Microbiol..

[b0175] Wang Y., Ouellette A.N., Egan C.W., Rathinavelan T., Im W., De Guzman R.N. (2007). Differences in the electrostatic surfaces of the type III secretion needle proteins PrgI, BsaL, and MxiH. J. Mol. Biol..

[b0180] Wang Y., Nordhues B.A., Zhong D., De Guzman R.N. (2010). NMR characterization of the interaction of the Salmonella type III secretion system protein SipD and bile salts. Biochemistry.

[b0185] Zhang Z., Marshall A.G. (1998). A universal algorithm for fast and automated charge state deconvolution of electrospray mass-to-charge ratio spectra. J. Am. Soc. Mass Spectrom..

[b0190] Zhang L., Wang Y., Picking W.L., Picking W.D., De Guzman R.N. (2006). Solution structure of monomeric BsaL, the type III secretion needle protein of *Burkholderia pseudomallei*. J. Mol. Biol..

[b0195] Zhang L., Wang Y., Olive A.J., Smith N.D., Picking W.D., De Guzman R.N., Picking W.L. (2007). Identification of the MxiH needle protein residues responsible for anchoring invasion plasmid antigen D to the type III secretion needle tip. J. Biol. Chem..

