# Supplementary material for: Reassessment of MxiH subunit orientation and fold within native Shigella T3SS needles using surface labelling and solid-state NMR
Source: J Struct Biol. 2015 Dec;192(3):441–8. doi: 10.1016/j.jsb.2015.10.005 (PMC4658334; doi:10.1016/j.jsb.2015.10.005)
Supplement: Supplementary material — Supplementary Figs. S1–S8 and Tables S1–S4. [file mmc1.pdf]

SUPPLEMENTARY MATERIAL FOR

Reassessment of MxiH subunit orientation and fold within native *Shigella* T3SS needles using surface labelling and solid-state NMR

Joeri Verasdonck[a,‡], Da-Kang Shen[b,‡], Alexander Treadgold[b,‡], Christopher Arthur[c], Anja Böckmann[d,*], Beat H. Meier[a,*] and Ariel J. Blocker[e,*]

[a]Physical Chemistry, ETH Zurich, 8093 Zurich, Switzerland.

[b]School of Cellular & Molecular Medicine, University of Bristol, BS8 1TD, Bristol, United Kingdom.

[c]School of Chemistry, University of Bristol, BS8 1TS, Bristol, United Kingdom.

[d]Institut de Biologie et Chimie des Protéines, Bases Moléculaires et Structurales des Systèmes Infectieux, Labex Ecofect, UMR 5086 CNRS, Université de Lyon, Lyon, France.

[e]Schools of Cellular & Molecular Medicine and Biochemistry, University of Bristol, BS8 1TD, Bristol, United Kingdom.

[‡]Joint first authors

[*]Corresponding authors: Ariel Blocker, ariel.blocker@bristol.ac.uk, tel +441173312063, fax +441173312091, Beat H. Meier, beme@ethz.ch; Anja Böckmann, a.bockmann@ibcp.fr

LIST OF SUPPLEMENTARY ITEMS

Table S1: *Shigella flexneri* strains used in this study
Table S2: Primer sequences used in this study
Table S3: Solid state NMR data collection conditions
Table S4: Assignment table
Figure S1: Location of the amino acids selected for mutagenesis in surface labelling experiments designed to test the differing MxiH models
Figure S2: Electrospray mass spectra of untreated and labelled needles
Figure S3: Sequence alignment showing variation between MxiH in *S. flexneri* serotype 5a and 6
Figure S4: 20-ms DARR spectrum of the natively-grown uniformly-labelled MxiH needles
Figure S5: Representative plane (^{13}C 59.54 ppm) of NCACB spectrum
Figure S6: 200-ms DARR spectrum
Figure S7: Representative plane (55.17 ppm) of the CCC-spectrum
Figure S8: Direct comparison of chemical shifts between the native needles assigned in this work and the *in vitro* polymerized needles by Demers et *al*. (2013).

TABLE 1. *Shigella flexneri* strains used in this study

Strain	Genotype (strain; plasmid)	Reference
WT	Wild-type M90T, serotype 5a	(Sansonetti et al., 1982)
Δ*mxiH*	SH116	(Blocker et al., 2001)
Δ*mxiH mxiH*	SH116; pACT3*mxiH*	(Shen et al., 2010)
Δ*mxiH* $mxiH_{L32C}$	SH116; pACT3$mxiH_{L32C}$	This study
Δ*mxiH* $mxiH_{V68C}$	SH116; pACT3$mxiH_{V68C}$	This study

TABLE 2. Primer sequences used in this study*

Primer	Sequence
MxiH_NdeI_For	ATTA<u>CATATG</u>AGTGTTACAGTACCGAATG
MxiH_HindIII_Rev	ATGC<u>AAGCTT</u>TTATCTGAAGTTTTGAATA
$MxiH_{L32C}$_For	GGTGAACTAACATGTGCACTAGATAAATTAGC
$MxiH_{L32C}$_Rev	GCTAATTTATCTAGTGCACATGTTAGTTCACC

*Underlined capital letters represent restriction endonuclease sites generated to facilitate cloning.

TABLE S3. Solid-state NMR data collection conditions*

Experiment	DARR 20ms	DARR 200ms	NCA	NCACB	CCC
Spectrometer	850 MHz	850 MHz	850 MHz	850 MHz	850 MHz
Probe	1.9 mm Bruker	1.9 mm Bruker	1.9 mm Bruker	1.9 mm Bruker	1.9 mm Bruker
MAS / kHz	18 kHz	18 kHz	18 kHz	18 kHz	18 kHz
Measurement Time	1.5d	1.5d	8h	5d	6d
Number of Scans	24	24	16	16	4
Interscan delay / s	2.6	2.6	3	2.5	2.6
**Transfer 1**	**HC-CP**	**HC-CP**	**HN-CP**	**HN-CP**	**HC-CP**
Time / ms	0.5	0.5	1	1	0.5
Field / kHz	50(C)/77(H)	50(C)/77(H)	50(N)/67.6(H)	50(N)/61.5(H)	50(C)/78(H)
Shape	tan(H) D=15 kHz	tan(H) D=15 kHz	tan(H) D=27 kHz	tan(H) D=25 kHz	tan(H) D=15 kHz
Carrier / ppm	63	63	123	121	49
**Transfer 2**	**DARR**	**DARR**	**N-CA**	**N-CA**	**DREAM**
Time / ms	20	200	6.5	6	4
Field / kHz	18(H)	18(H)	13.5(N)/4.7(C)	13.5(N)/4.7(C)	8.7(C)
Shape	-	-	tan(C) D=1.9 kHz	tan(C) D=1.9 kHz	tan(C) D=3.5 kHz
Carrier / ppm	-	-	50	50	52
**Transfer 3**	-	-	-	**DREAM**	**DARR**
Time / ms				4	80
Field / kHz				8.7(C)	18(H)
Shape				tan(C) D=3.5 kHz	-
Carrier / ppm				52	-
**t1 Increments**	2048	2048	600	95	220
Sweep width / kHz	60	60	24.4	5	85
carrier / ppm	110	110	123	121	50
acq. time / ms	17.1	17.1	12.3	9.5	6.1
zero-filling points	4096	4096	2048	256	256
window function	qsine 3	qsine 3	qsine 3	qsine 2.6	qsine 2.6
**t2 Increments**	3072	3072	3072	110	110
Sweep width / kHz	100	100	81.5	8.3	85
carrier / ppm	81.5	81.5	54	50	50
acq. time / ms	18.8	18.8	18.8	6.6	6.1
zero-filling points	8192	8192	8192	256	256
window function	qsine 3	qsine 3	qsine 3	qsine 2.6	qsine 2.6
**t3 Increments**	-	-	-	2560	3072
Sweep width / kHz				81.5	380
carrier / ppm				50	96
acq. time / ms				15.7	18.8
zero-filling points				4096	4096
window function				2.6	2.6
**Decoupling**	SPINAL64	SPINAL64	SPINAL64	SPINAL64	SPINAL64
Field / kHz	90	90	90	90	90

*The value "D" for the shape refers to the difference between minimum and maximum B_1 frequency

TABLE S4. Assignment table

1	Met	N		CA		CO		CB													
2	Ser	N		CA	57,61	CO	170,31	CB	63,16												
3	Val	N	121,56	CA	61,45	CO	174,89	CB	34,27	CG1	18,55	CG2	20,83								
4	Thr	N	127,29	CA	61,97	CO	174,36	CB	68,28	CG2	22,44										
5	Val	N	125,61	CA	68,65	CO	175,45	CB	28,77	CG1	23,33	CG2	21,83								
6	Pro	N	133,91	CA	65,31	CO	175,98	CB	31,90	CD	49,64	CG	27,28								
7	Asn	N	118,56	CA	52,36	CO	176,82	CB	39,15	CG	175,27										
8	Asp	N	116,62	CA	52,36	CO	176,80	CB	40,94	CG	180,66										
9	Asp	N	112,87	CA	54,12	CO	176,82	CB	41,18	CG	180,82										
10	Trp	N	119,82	CA	54,83	CO	175,55	CB	30,33	CD1	128,82	CD2	130,80	CE2	138,97	CG	109,55	CZ2	112,99	CZ3	120,42
11	Thr	N	110,14	CA	58,40	CO	176,06	CB	73,44	CG2	21,05										
12	Leu	N	120,44	CA	58,44	CO	179,28	CB	42,05	CD1	26,53	CG	26,96								
13	Ser	N	114,50	CA	63,04	CO	175,78	CB	62,47												
14	Ser	N	123,82	CA	61,77	CO	178,54	CB	62,17												
15	Leu	N	128,27	CA	58,49	CO	178,05	CB	41,44	CD1	27,19	CD2	25,28	CG	27,38						
16	Ser	N		CA	62,64	CO	180,52	CB	62,92												
17	Glu	N	119,40	CA	58,88	CO	177,77	CB	29,88	CD	184,08	CG	36,53								
18	Thr	N	116,01	CA	67,26	CO	178,11	CB	68,81	CG2	20,78										
19	Phe	N	120,36	CA	61,46	CO	177,03	CB	38,43	CD1	132,64	CD2	132,64	CE1	130,31	CE2	130,31	CG	138,97		
20	Asp	N	120,73	CA	57,57	CO	177,72	CB	40,12	CG	178,51										
21	Asp	N	119,46	CA	56,84	CO	180,15	CB	40,53	CG	178,30										
22	Gly	N	107,72	CA	46,98	CO	175,31														
23	Thr	N	105,31	CA	60,54	CO	175,97	CB	69,81	CG2	19,91										
24	Gln	N	123,55	CA	60,27	CO	179,72	CB	28,58	CD	180,30	CG	34,30								
25	Thr	N	118,75	CA	67,16	CO	176,36	CB	67,49	CG2	21,93										
26	Leu	N	126,27	CA	58,30	CO	178,51	CB	41,17	CD1	24,77	CD2	23,48	CG	28,64						
27	Gln	N	116,60	CA	61,46	CO	179,03	CB	30,31	CD	177,70	CG	37,45								
28	Gly	N	110,26	CA	46,94	CO	177,16														
29	Glu	N	123,91	CA	59,18	CO	180,72	CB	29,33	CD	183,05	CG	36,74								
30	Leu	N	122,71	CA	57,77	CO	177,11	CB	41,89	CD1	26,28	CG	26,91								
31	Thr	N	117,86	CA	67,33	CO	176,52	CB	69,15	CG2	22,61										
32	Leu	N	118,05	CA	57,73	CO	179,13	CB	41,89	CD1	23,30	CG	25,72								
33	Ala	N	121,95	CA	55,02	CO	178,95	CB	17,87												
34	Leu	N		CA	58,30	CO		CB	42,14	CD1	25,35	CG	27,32								
35	Asp	N	120,33	CA	57,25	CO	178,2	CB	42,10	CG	179,81										
36	Lys	N	115,20	CA	59,58	CO	179,41	CB	33,62	CD	30,20	CE	41,92	CG	26,13						

37	Leu	N	123,55	CA	56,60	CO	178,13	CB	42,47	CD1	25,41	CD2	26,59	CG	26,61						
38	Ala	N	118,85	CA	54,02	CO	177,35	CB	17,48												
39	Lys	N	110,00	CA	55,92	CO	177,27	CB	34,25	CD	29,42	CG	25,80								
40	Asn	N		CA	50,35	CO	171,45	CB	38,37												
41	Pro	N		CA	62,77	CO	174,68	CB	31,26	CG	27,29										
42	Ser	N	106,99	CA	57,10	CO	174,83	CB	67,26												
43	Asn	N	126,48	CA	51,70	CO	172,77	CB	39,42	CG	176,80										
44	Pro	N	139,21	CA	65,42	CO	176,92	CB	32,92	CD	51,07	CG	27,47								
45	Gln	N	116,22	CA	58,22	CO		CB	26,97	CG	32,14										
46	Leu	N	121,48	CA	58,01	CO	178,33	CB	41,97	CD1	25,37	CG	27,75								
47	Leu	N	117,94	CA	58,33	CO		CB	42,02	CD1	24,73	CD2	23,56	CG	26,25						
48	Ala	N	122,96	CA	55,27	CO	182,04	CB	18,58												
49	Glu	N	122,29	CA	59,12	CO	179,10	CB	29,20	CG	36,65										
50	Tyr	N	119,82	CA	62,59	CO	176,41	CB	38,48												
51	Gln	N	117,04	CA	60,19	CO	180,76	CB	27,25	CD	179,21	CG	32,94								
52	Ser	N	116,19	CA	63,42	CO	175,63	CB	62,06												
53	Lys	N	122,32	CA	58,32	CO	178,86	CB	32,11	CD	27,42	CE	42,82	CG	24,85						
54	Leu	N	120,69	CA	57,80	CO	179,48	CB	40,87	CD1	21,47	CD2	25,21	CG	26,35						
55	Ser	N	113,12	CA	63,20	CO	176,01	CB	63,61												
56	Glu	N	121,94	CA	61,46	CO	177,50	CB	31,31	CD	182,33	CG	39,26								
57	Tyr	N	119,18	CA	62,68	CO	176,95	CB	39,26	CD1	132,27	CD2	132,27	CE1	118,17	CE2	118,17	CG	130,55		
58	Thr	N	111,18	CA	66,09	CO	176,34	CB	68,62	CG2	21,89										
59	Leu	N	123,89	CA	57,75	CO	178,42	CB	40,22	CD1	22,13	CD2	27,97	CG	26,21						
60	Tyr	N	124,11	CA	59,01	CO	176,93	CB	36,23	CD1	134,27	CD2	134,27	CE1	118,17	CE2	118,17	CG	129,50	CZ	157,73
61	Arg	N	117,52	CA	56,61	CO	180,50	CB	30,94	CD	42,27	CG	25,39	CZ	159,57						
62	Asn	N	119,31	CA	56,96	CO	176,07	CB	39,92	CG	175,13										
63	Ala	N	123,98	CA	56,05	CO	180,07	CB	17,49												
64	Gln	N	121,93	CA	57,97	CO	175,70	CB	31,30	CD	178,29	CG	32,91								
65	Ser	N	109,93	CA	60,58	CO	179,38	CB	63,50												
66	Asn	N	117,36	CA	55,85	CO	177,05	CB	37,21	CG	174,02	ND2	109,91								
67	Thr	N	116,66	CA	68,98	CO	174,63	CB	68,72	CG2	20,93										
68	Val	N	120,71	CA	67,27	CO	176,52	CB	32,13	CG1	22,54	CG2	20,81								
69	Lys	N	118,84	CA	58,40	CO	177,10	CB	32,56	CD	28,02	CE	42,44	CG	25,42						
70	Val	N	118,72	CA	66,90	CO	179,72	CB	31,87	CG1	24,11	CG2	22,16								
71	Ile	N	117,95	CA	61,52	CO	177,62	CB	34,30	CD1	7,68	CG1	26,85	CG2	17,64						
72	Lys	N	123,67	CA	61,33	CO	178,42	CB	30,67	CD	29,38	CE	42,04	CG	24,88						
73	Asp	N	118,85	CA	57,46	CO	180,73	CB	39,34	CG	179,25										
74	Val	N	124,71	CA	66,32	CO	176,78	CB	31,82	CG1	25,07	CG2	21,48								
75	Asp	N	119,57	CA	55,20	CO	177,22	CB	38,00	CG	172,18										
76	Ala	N	121,28	CA	54,73	CO	180,00	CB	18,11												
77	Ala	N	121,29	CA	54,71	CO	180,01	CB	18,07												

78	Ile	N	119,97	CA	64,99	CO	178,33	CB	38,22	CD1	15,26	CG1	28,13	CG2	18,48				
79	Ile	N	116,82	CA	63,44	CO	180,00	CB	38,02	CD1	15,11	CG1	31,57	CG2	17,15				
80	Gln	N	119,84	CA	57,93	CO	177,09	CB	28,19	CD	180,68	CG	33,52						
81	Asn	N	115,31	CA	52,60	CO	175,98	CB	37,74	CG	175,38								
82	Phe	N	118,30	CA	56,58	CO	175,21	CB	38,16	CD1	129,35	CD2	129,35	CE1	129,76	CE2	129,76	CG	140,28
83	Arg	N	117,27	CA	57,13	CO	181,45	CB	31,79	CD	43,53	CG	27,51	CZ	159,56				

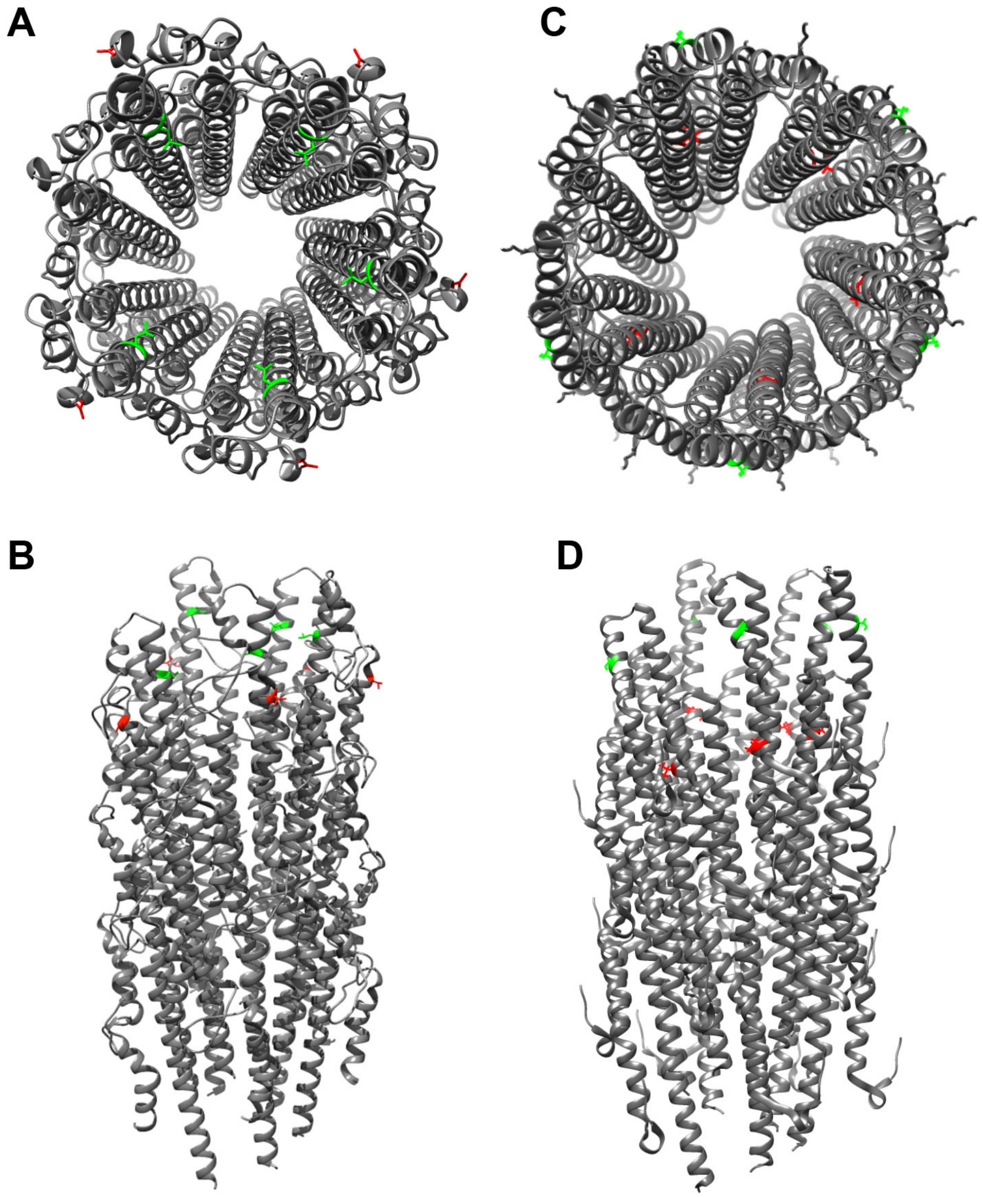

Figure S1. Location of the amino acids selected for mutagenesis in surface labelling experiments designed to test the differing MxiH models. A) and B) Top and side view of 22-mer of Fujii *et al.* (2012) model (PDB IB 2J0R), respectively. C) and D)Top and side view of 22-mer of Demers *et al.* (2014) model (PDB IB 2MME), respectively. Amino acids selected for mutagenesis to cysteines are shown as stick models only in the top five MxiH subunits of each model. L32 is in green and V68 in red.

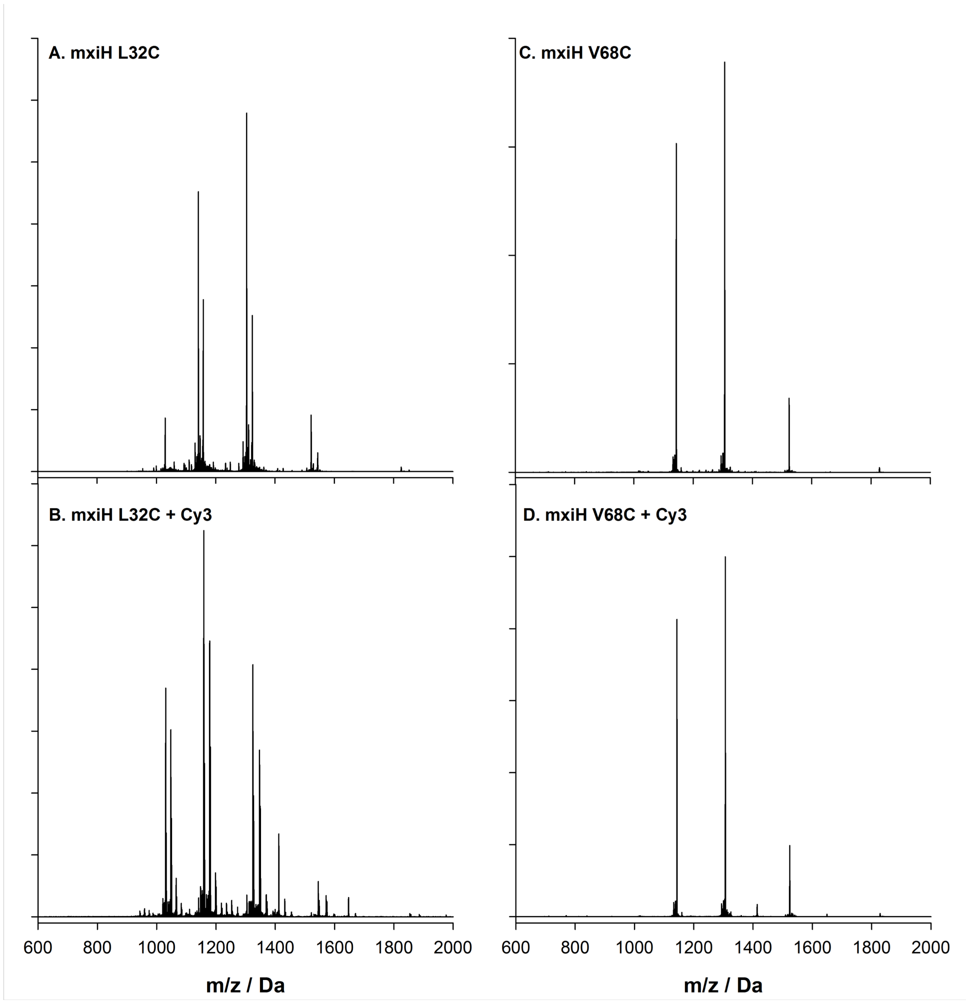

Figure S2. Electrospray mass spectra of untreated and labelled needles. Native mass spectrometry analysis of purified needles (A and C) and purified needles labelled with Cy3 maleimide (B and D). Spectra were recorded over mass-to-charge ratio (m/z) window of 600-2000.

```
MxiH serotype 5a
MxiH serotype 6

         10         20         30         40         50

MSVTVPNDDW TLSSLSETFD DGTQTLQGEL TLALDKLAKN PSNPQLLAEY
MSVTVPDKDW TLSSLSETFD DGTQTLQGQL TSALNALAEN PSNPQLLAEY
      **                       *   *  **  *
         60         70         80
QSKLSEYTLY RNAQSNTVKV IKDVDAAIIQ NFR
QSKLSEYTLY RNAQSNTVKV IKDVDAAIIQ NFR
```

Figure S3. Sequence alignment showing variation between MxiH in *S. flexneri* serotypes 5a and 6. Individual sequences are shown using the same colour code as in **Fig. 5** and varying amino acids are indicated by red asterisks.

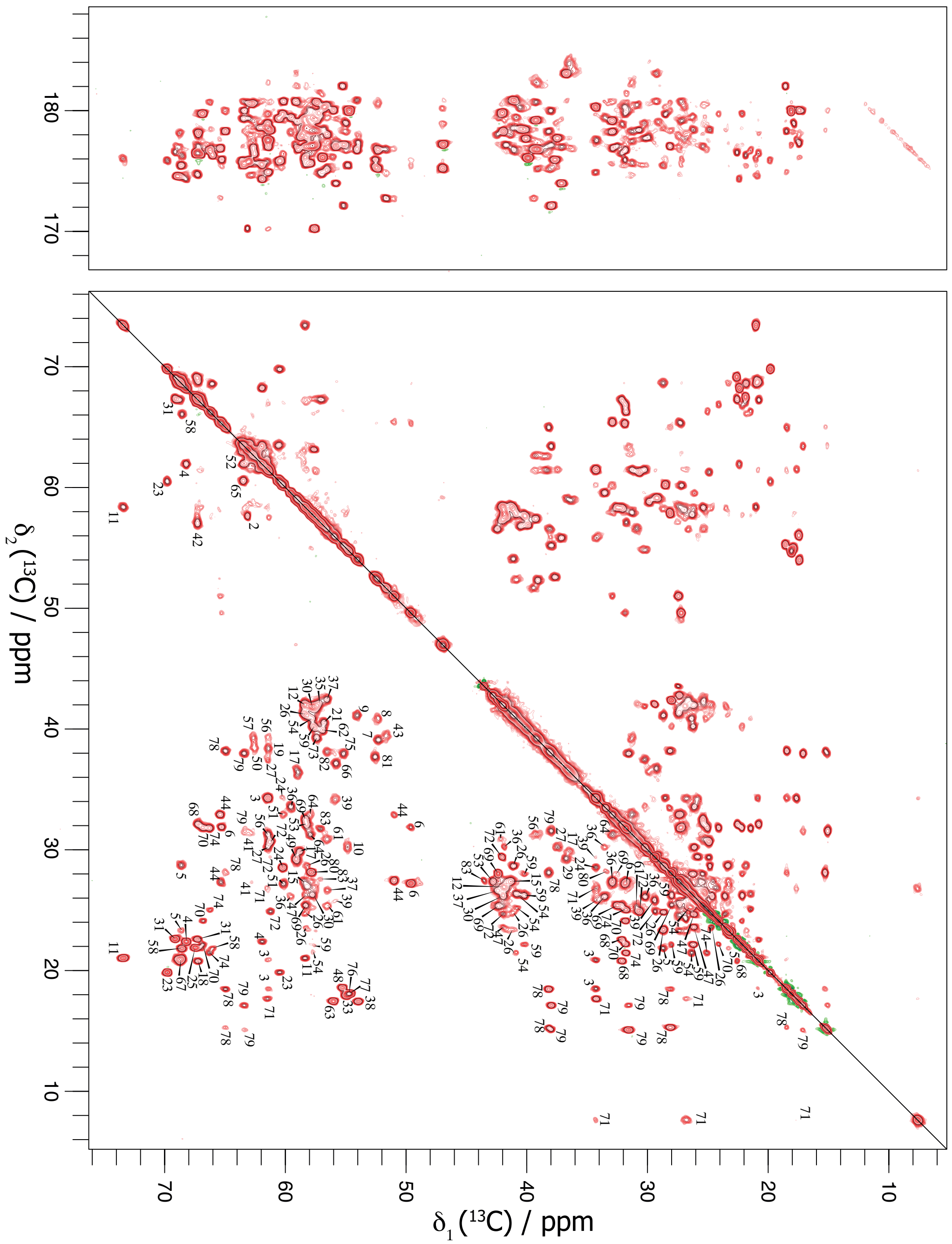

Figure S4. 20-ms DARR spectrum of the natively-grown uniformly-labelled MxiH needles. The data has been zero-filled and apodized with a shifted squared sine-bell function. Assigned peaks have been labelled on one side of the diagonal.

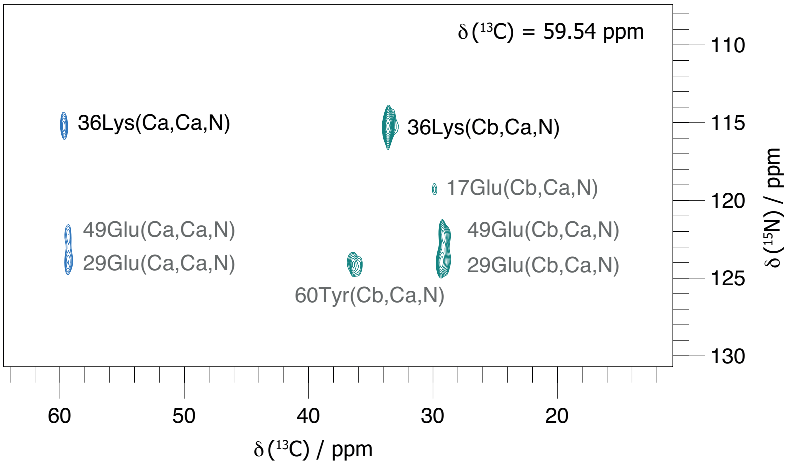

Figure S5. Representative plane (^{13}C 59.54 ppm) of NCACB spectrum. Black labels indicate peaks with their maximum in this plane. Grey labels indicate peaks with their maximum in another plane.

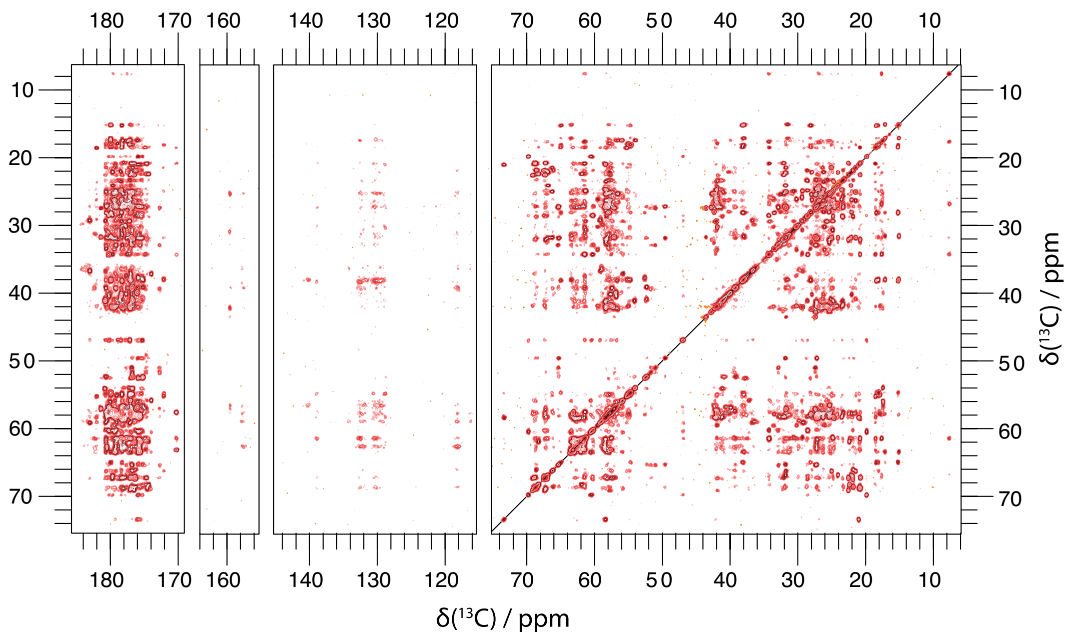

Figure S6. 200-ms DARR spectrum. The data has been zero-filled and apodized with a shifted squared sine-bell function.

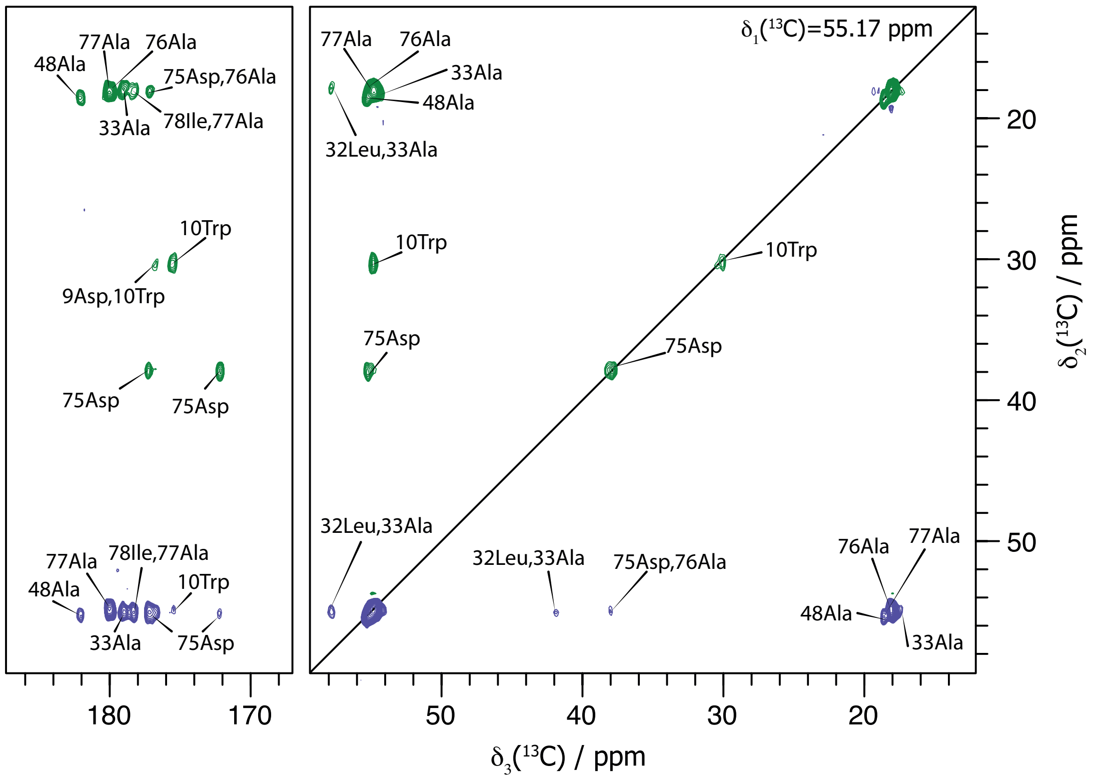

Figure S7. Representative plane (55.17 ppm) of the CCC-spectrum. This contains a number of sequential contacts that were used to properly assign serotype-specific mutations. The data has been zero-filled and apodized with a shifted squared sine-bell function.

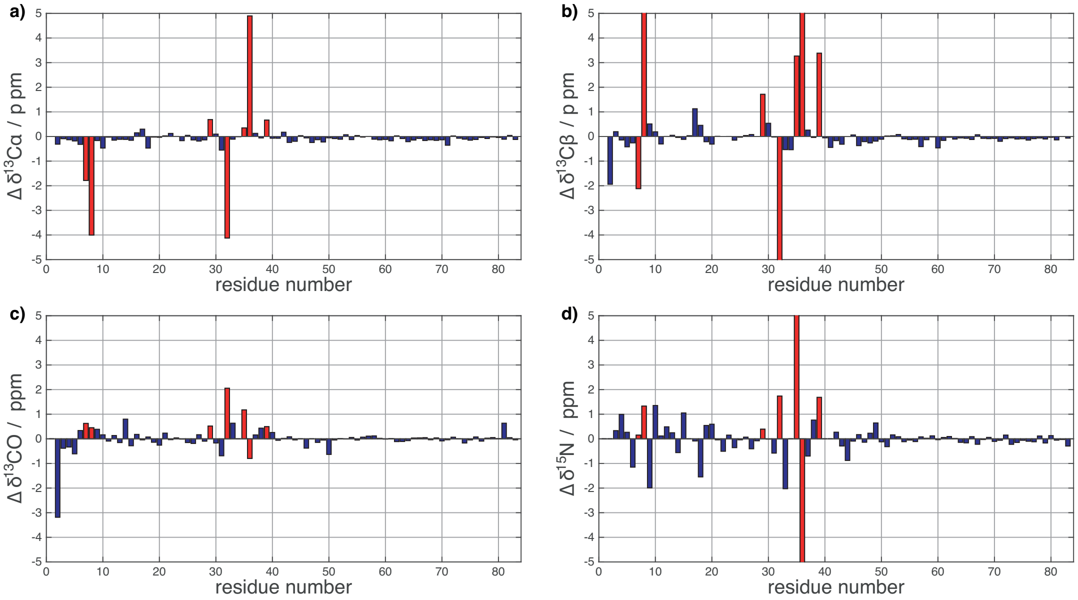

Figure S8. Direct comparison of chemical shifts between the native needles assigned in this work and the *in vitro* polymerized needles by Demers et *al*. (2013). Comparisons are made for Cα **(a)** , Cβ **(b)**, CO **(c)** and N **(d)**. Red bars highlight the serotype differences in the sequence. The rather large deviation of Ser2 can be explained by the presence of non-native Gly and His at the N-terminus in the work of Demers et *al*.
